# Workplace violence against nursing interns and patient safety: The multiple mediation effect of professional identity and professional burnout

**DOI:** 10.1002/nop2.1560

**Published:** 2022-12-25

**Authors:** Qianqian Yang, Linlin Yang, Chunling Yang, Xia Wu, Yue Chen, Pingping Yao

**Affiliations:** ^1^ Medical School, Liaocheng University Liaocheng China; ^2^ Nursing Department of Liaocheng People's Hospital Liaocheng China; ^3^ Nursing Department of Taian City Central hospital Taian China

**Keywords:** burnout, graduate, internship, nursing, patient safety, professional identity, serial multiple mediation, workplace violence

## Abstract

**Background:**

Most nursing interns have suffered some form of workplace violence in clinical settings, which has been linked to the jeopardizing of patient safety. Although previous research studies have examined the effect of workplace violence on patient safety, few studies have examined whether workplace violence is associated with patient safety through professional identity and professional burnout among nursing interns.

**Aims:**

To test whether professional identity and professional burnout play mediating roles in the relationship of workplace violence and patient safety among nursing interns.

**Design:**

Cross‐sectional study.

**Methods:**

The study included 466 nursing interns from three tertiary grade A hospitals. The Workplace Violence Scale, the Professional Identity Scale, the Maslach Burnout Inventory‐General Survey, and the Patient Safety Behaviour Scale were used to gather data. Associations among workplace violence, professional identity, professional burnout, and patient safety were assessed by correlation and the serial‐multiple mediation analysis.

**Results:**

Workplace violence, professional identity, professional burnout and patient safety were significantly correlated. Workplace violence can have a direct positive impact on patient safety of nursing interns, but also an indirect impact on patient safety through three paths: the independent mediating role of professional identity, the independent mediating role of professional burnout, and the chain mediating role of professional identity and professional burnout.

**Conclusions:**

Our findings suggest that workplace violence can affect patient safety through decreasing professional identity and increasing professional burnout among nursing interns. Interventions aimed at decreasing workplace violence among nursing interns would be beneficial for professional attitude and patient safety.

## 1 BACKGROUND

Patient safety is one of the most important underlying principles in the provision of health services. However, providing safe care is one of the biggest challenges in hospital management (World Health Organization [WHO], 2017). The WHO (2018) states that patient harm is the 14th principal cause of the global disease burden. Some studies have shown that 1 in 10 patients is injured during the therapeutic process, and about 43 million patient safety‐related adverse events happen annually (Liu et al., 2019). Unsafe care can increase morbidity and mortality, medical expenses, disability, nosocomial infections, and low healthcare efficiency (Song & Guo, 2019).

Searching for the aspects that contribute to patient safety is of utmost importance in health care systems. Workplace violence refers to incidents in which staff are threatened, assaulted, or abused in circumstances associated with their work, including physical assault, verbal abuse or threats, bullying, sexual harassment, and aggression (Liu et al., 2019). A growing body of literature has linked workplace violence to patient safety. Liu et al. (2019) analysed data collected from 1502 nurses working in 23 hospitals in China and demonstrated that workplace violence against nurses was linked to decreased patient safety and more adverse events. Roche et al. (2010) conducted a cross‐sectional study collecting data on 94 nursing wards in 21 hospitals and demonstrated that violence toward nurses could delay tasks and increase medical errors. However, most existing studies focus on clinical nurses, with only limited attention to nursing interns.

As new members of the nursing staff in hospitals, nursing interns provide direct basic care to patients and spend much of their work time with patients; they therefore play a very important role in patient safety (Cheung et al., 2019). However, when starting their clinical practice, nursing interns are unfamiliar with hospital environments, unskilled in operation, and lack theoretical knowledge and communication skills; these limitations could pose occupational safety hazards (Gorgich et al., 2016; Song & Guo, 2019; Stevanin et al., 2015). Previous studies have shown that approximately 17%–53.2% of nursing interns reported adverse nursing safety events (Stevanin et al., 2018), although most are not reported (Stevanin et al., 2018). Contrastingly, few nursing interns accept training for preventing and coping with workplace violence. Hospitals and nursing schools neglect workplace violence against nursing interns, which leads to a high prevalence of workplace violence against nursing interns. In an US study, over 50% of nursing interns reported experiencing horizontal violence at least once during a clinical setting (Wallace & Tucker, 2019). Cheung et al. (2019) found that 30.6% of nursing interns in Hong Kong reported having received verbal threats, and 16.5% reported having been physically assaulted. Different from clinical nurses who have already had a relatively stable nursing career, nursing interns are still considering whether they will follow nursing as a career. Considering nursing interns, it is unclear whether workplace violence affects patient safety. Likewise, few studies have suggested strategies that reduce workplace violence events among nursing interns.

Exploring the link between workplace violence toward nursing interns and patient safety may provide a fresh perspective for identifying risk factors and undertaking effective measures to improve patient safety (Liu et al., 2019). Professional identity has been established as an important theme in the research on nursing interns; it refers to the professional self, self‐concept, and working ability of nurses and represents how nursing students and nurses perceive the nursing profession (Johnson et al., 2012). According to past studies, a low level of professional identity could affect healthcare workers' quality of medical services and increase patient safety events (Chen, 2016; Qiu et al., 2019). On the contrary, an intervention study indicated that developing a nursing professional identity promotion program could reduce the incidence of patient safety events (Heldal et al., 2019). In addition, some empirical studies have found that workplace violence among medical staff can decrease the level of professional identity (Arnetz & Arnetz, 2001; Qiu et al., 2019). Considering the associations between professional identity, workplace violence, and patient safety, a higher frequency of workplace violence may decrease nurses' level of professional identity and further increase adverse patient‐related events. Thus, one aim of the current study is to verify whether professional identity mediates the influence of workplace violence on patient safety for nursing interns.

Previous research studies supported the viewpoint that professional burnout may mediate the relationship between workplace violence and patient safety. Professional burnout refers to a syndrome characterized by emotional exhaustion, depersonalization, and reduced sense of personal achievement (Maslach et al., 2001). Based on the job demands–resources model, professional demands—such as high working pressure, unfavourable work environment, and interpersonal conflicts—may result in professional dissatisfaction and burnout and negatively influence employees' professional performance (Bakker & Demerouti, 2007; Nakagawa et al., 2014). Liu et al. (2019) showed that nurse‐reported workplace violence was associated directly with higher incidences of burnout, lower patient safety, and more adverse events, and that workplace violence had an indirect effect on patient safety through the mediating effect of burnout. Boafo and Hancock (2017) found that nurses who had suffered physical assaults were 2.7 times more likely to leave the nursing profession, and professional burnout was associated with unsafe patient care (Liu et al., 2019). However, few studies have explored the mediating role of professional burnout in the association between workplace violence and patient safety among nursing interns.

Furthermore, professional identity could be considered as an important influencing factor for professional burnout (Zhang et al., 2018). For example, Zhang, Feng, et al. (2021) conducted a national survey and found that professional identity was negatively associated with burnout and turnover intention, that is, as professional identity decreased, professional burnout increased. Additionally, the effect of professional identity on turnover intention was mediated by burnout. Jung and Yoo (2020) demonstrated that participating in a career efficacy enhancement program (i.e., a professional identity accelerated program), is helpful in reducing the practical problems caused by professional burnout. Therefore, we hypothesize that professional identity and burnout have a serial mediation effect on the relationship between workplace violence and patient safety among nursing interns.

In summary, collective evidence supports the negative effects of workplace violence on professional identity, professional burnout, and patient safety. However, researchers failed to examine the effects of workplace violence on patient safety among nursing interns. Furthermore, no studies have focused on the examination of either the direct or indirect effect of workplace violence on patient safety. Specifically, no study has documented the mediating effects of professional identity and professional burnout in this relationship. Therefore, the current study aimed to investigate the nature of the association between workplace violence and patient safety, and whether professional identity and professional burnout mediate this relationship among nursing interns. As such, we propose the following three hypotheses:

Hypothesis 1 Professional identity uniquely (independent of professional burnout) mediates the relationship between workplace violence and patient safety among nursing interns.

Hypothesis 2 Professional burnout uniquely (independent of professional identity) mediates the relationship between workplace violence and patient safety among nursing interns.

Hypothesis 3 Professional identity and professional burnout co‐play a serial mediating role in the relationship between workplace violence and patient safety among nursing interns. The sequence of the pathway will be: workplace violence → professional identity → professional burnout → patient safety.

## 2 METHODS

### 2.1 Study design and participants

We used a cross‐sectional design and collected survey data from nursing interns. Participants were recruited from three general hospitals in Shandong Province, China, from October to December 2021, using a convenience sampling method. The researchers contacted the nursing managers of the three hospitals to obtain permission. Before completing the questionnaires, participants were asked to read the study instructions carefully, and were informed that their participation was entirely voluntary and that not participating would not affect their evaluations. Researchers then invited all willing and eligible participants to complete online questionnaires. The inclusion criteria were that the nursing intern (1) was older than 18 years and (2) was in the process of doing their internship for at least 6 months. The exclusion criteria were (1) having been diagnosed with psychological or mental disorders, and (2) being absent because of vacation, illness, or deployment in a non‐nursing work unit for at least 1 month. We computed the sample size based on the sample size equation *n* = *Z* ^2^_α/2_ *P*(1‐*P*)/*δ* ^2^ (Zhou et al., 2012), assuming a type I error (*α*) of 0.05, and *Z*_α/2_ was set at 1.96. For prevalence (*P*), we set a reference value of 11.93%, consistent with a previous study that investigated the percentage of nursing safety events reported by nursing interns, which was 11.93% (Chen & Shen, 2020). We used 0.03 for the absolute error (*δ*). A sample size of 449 was derived. Considering a 10% dropout rate, 499 nursing interns were screened for the study. Furthermore, of the distributed questionnaires, 466 correctly completed questionnaires were collected—a response rate of 93.2%.

### 2.2 Data collection

All participants completed a questionnaire containing standardized survey questions. Demographic information, workplace violence, professional identity, professional burnout, and patient safety were obtained using anonymous self‐reported structured questionnaires.

### 2.3 Measures

#### 2.3.1 Demographic characteristics

Demographic characteristics included age, sex, ethnicity, level of education, current department, and location, as well as whether they were an only child in the family, class officers, or healthcare workers among family members. Ethnicity was categorized as Han or other nationalities. Their location was categorized as urban or rural. Education level was categorized as junior college or undergraduate. Current department was measured by an item on “which department are you interning in now?” (medical department, surgical department, ICU, paediatrics department, obstetric department, gynaecological department, emergency department, operating room, or others).

#### 2.3.2 Workplace violence

The prevalence of workplace violence was assessed using the Chinese version of the Workplace Violence Scale (WVS). The scale was developed by Liu et al. (2019) based on the WHO's definition of “workplace violence.” It evaluates nursing interns' experiences of workplace violence for the previous 6 months. The WVS comprises six items. Each item is scored on a four‐point scale reflecting the respondents' frequency of exposure to workplace violence (0 = 0 times, 1 = once, 2 = 2 or 3 times, 3 = more than 3 times). The total possible score is the sum of each item, ranging from 0 to 18, with a higher total score indicating a higher frequency of exposure to workplace violence. The Cronbach's *α* value of the scale was 0.876 (Li, 2019). In the current study, the Cronbach's *α* for the WVS was 0.802.

#### 2.3.3 Professional identity

The level of professional identity of nursing interns was evaluated using the Professional Identity Scale (PIS), developed by Liu et al. (2011), with satisfactory credibility. The Cronbach's *α* for the PIS was 0.938, and the split‐half reliability was 0.88 (Liu et al., 2011). This instrument consists of 30 items and five subscales: professional identity evaluation, professional social support, professional social proficiency, dealing with professional frustration, and professional self‐reflection. Each item was ranked on a five‐point Likert scale from 1 = strongly disagree to 5 = strongly agree. The total score ranged from 30 to 150, with higher scores indicating a higher level of professional identity. The PIS displayed good reliability and validity in previous studies (Zhang, Feng, et al., 2021; Zhang, Zuo, et al., 2021) and good internal consistency in the current study (Cronbach's *α* = 0.975).

#### 2.3.4 Professional burnout

Professional burnout was measured using the Chinese version of the Maslach Burnout Inventory‐General Survey (MBI‐GS) (Li & Liu, 2000). The MIB‐GS consists of 22 items and three subscales: emotional exhaustion (EE), depersonalization (DP), and personal accomplishment (PA). Each item was rated on a seven‐point Likert scale, (ranging from 0 = never) to 6 = every day. The EE and DP items were positively scored. The PA items were reversed scored, and finally, the subscale was labelled as reduced personal accomplishment. The total score of the MBI‐GS ranged from 0 to 132, with higher scores indicating a high level of professional burnout. A Cronbach's *α* value of 0.93 was reported for professional burnout (Li & Liu, 2000). In the current study, the MBI‐GS displayed good internal consistency (Cronbach's *α* = 0.899).

#### 2.3.5 Patient safety

The Patient Safety Behaviour Scale (PSBS) for nursing students developed by Shih in 2008 was used to measure patient safety (Shih et al., 2008). Nursing students were asked to rate the frequency of these patient safety‐related adverse events. The PSBS comprised 12 items, with each item ranked on a five‐point Likert scale from 1 = never to 5 = always. The total PSBS score ranged from 12 to 60, with higher scores indicating better patient safety‐related behaviour. It has a Cronbach's *α* of 0.889 (Shih et al., 2008). The PSBS displayed good internal consistency in the current study (Cronbach's *α* = 0.860).

### 2.4 Statistical analysis

This study used IBM SPSS Statistics (version 20.0) for data analysis. First, a descriptive analysis was employed to describe the demographic characteristics, workplace violence, professional identity, professional burnout, and patient safety of the study population. Second, a Pearson's correlation coefficient was used to determine whether workplace violence, professional identity, professional burnout, and patient safety were correlated among each other. Finally, to determine whether there were multiple mediation effects of professional identity and professional burnout between workplace violence and patient safety, we used IBM SPSS Statistics (version 20.0) PROCESS (Model 6) designed by Igartua (Igartua & Hayes, 2021) to conduct a data analysis. The indirect effect and bias‐corrected confidence intervals (CIs), which were estimated using PROCESS, were based on 5000 bootstrapping samples. If the 95% CIs did not include zero, the mediating effect was considered significant. The PIS and MBI‐GS scores were entered as mediators, the WVS score was entered as the independent variable, and the PSBS score was used as the dependent variable.

## 3 RESULTS

### 3.1 Demographic characteristics of sample

Table 1 lists the demographic characteristics, as well as the results on workplace violence, professional identity, professional burnout, and patient safety of the study sample. Data were collected from 466 nursing interns and the average age of the sample was 20.26 (±1.07) years (mean [±SD]). Most were female (83.48%), ethnic Han (98.93%), and rural (79.61%). Of the full sample, 90 (19.31%) participants were only children and 149 (31.97%) were class officers. Of the 466 interns, 87.55% were attending junior college and 12.45% undergraduate school. For 2.36% of the interns, their family members had medical backgrounds.

**TABLE 1:** Demographic characteristics, workplace violence, professional identity, professional burnout, and patient safety among nursing interns (*N* = 466)

Variables	Category	Mean (±SD)/*n* (%)
Demographic characteristics		
Age (years)		20.26 (±1.07)
Sex	Male	77 (16.52)
	Female	389 (83.48)
Ethnic	Han	461 (98.93)
	Others	5 (1.07)
Location	Urban	95 (20.39)
	Rural	371 (79.61)
Only child	Yes	90 (19.31)
	No	376 (80.69)
Class officer	Yes	149 (31.97)
	No	317 (68.03)
Level of education	Junior College	408 (87.55)
	Undergraduate	58 (12.45)
Healthcare workers among family members	Yes	11 (2.36)
	No	455 (97.64)
Current Department	Medical Department	162 (34.76)
	Surgical Department	137 (29.40)
	ICU	42 (9.01)
	Paediatrics Department	34 (7.30)
	Obstetric Department	30 (6.44)
	Gynaecological Department	18 (3.86)
	Emergency Department	13 (2.79)
	Operating room	23 (4.94)
	Others	7 (1.50)
Workplace violence		6.61 (±1.42)
Professional identity		120.15 (±20.74)
Professional burnout		51.75 (±22.96)
Patient safety		55.63 (±5.39)

*Note*: The median and interquartile (P25–P75) of age was 20 (20–21). The median and interquartile (P25–P75) of workplace violence was 6 (6–7). The median and interquartile (P25–P75) of professional identity was 120 (106.75–140). The median and interquartile (P25–P75) of professional burnout was 47 (34–69). The median and interquartile (P25–P75) of patient safety was 58 (53–60).

### 3.2 Correlation of workplace violence, professional identity, professional burnout, and patient safety

The correlations between workplace violence, professional identity, professional burnout, and patient safety are presented in Table 2. The results showed that workplace violence was negatively related to professional identity (*r* = −0.257, *p* = 0.000) and patient safety (*r* = −0.249, *p* =0.000) and positively related to patient burnout (*r* = 0.321, *p* = 0.000). Professional identity was negatively related to professional burnout (*r* = −0.602, *p* = 0.000) and positively related to patient safety (*r* = 0.492, *p* = 0.000). Professional burnout was negatively related to patient safety (*r* = −0.454, *p* = 0.000). The *p*‐values of all the analysis results were significant at *p* < 0.01 (two‐tailed).

**TABLE 2:** Correlations between workplace violence, professional identity, professional burnout and patient safety

Variables	Workplace violence		Professional identity		Professional burnout		Patient safety	
	*r*	*p*	*r*	*p*	*r*	*p*	*r*	*p*
Workplace violence	1							
Professional identity	−0.257	0.000	1					
Professional burnout	0.321	0.000	−0.602	0.000	1			
Patient safety	−0.249	0.000	0.492	0.000	−0.454	0.000	1	

*Note*: *p*‐values based on Pearson's correlation.

### 3.3 Mediation analysis of professional identity and professional burnout

**FIGURE 1:**
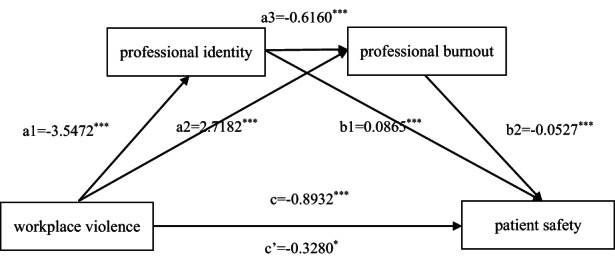
Serial mediation models for workplace violence, professional identity, professional burnout, and patient safety. *Note*: *p < 0.05, **p < 0.01, and ***p < 0.001.

The serial mediation analysis results of the impact of professional identity and burnout on workplace violence and patient safety are shown in Figure 1 and Table 3. The total effects of workplace violence on patient safety, shown in Figure 1 and Table 3, were significant (*p* < 0.001), indicating a higher frequency of workplace violence and a higher likelihood of patient safety issues.

**TABLE 3:** Hypothesized serial mediation model of professional identity and professional burnout between workplace violence and patient safety

Pathway	Effect	SE	BootLLCI	BootULCI
Total effect (c)	−0.8932	0.1614	−1.2103	−0.5761
Direct effect (c')	−0.3280	0.1492	−0.6212	−0.0347
a1	−3.5472	0.6197	−4.7649	−2.3295
a2	2.7182	0.5730	1.5923	3.8442
a3	−0.6160	0.0415	−0.6975	−0.5345
b1	0.0865	0.0128	0.0613	0.1117
b2	−0.0527	0.0118	−0.0759	−0.0294
Indirect effects				
Total indirect effects	−0.5652	0.1338	−0.8575	−0.3506
Indirect 1	−0.3070	0.1282	−0.6495	−0.1399
Indirect 2	−0.1151	0.0432	−0.2246	−0.0456
Indirect 3	−0.1432	0.0762	−0.3338	−0.0331

*Note*: Indirect 1, workplace violence → professional identity → patient safety; Indirect 2, workplace violence → professional identity → professional burnout → patient safety; Indirect 3, workplace violence → professional burnout → patient safety.

Abbreviations: BootLLCI, bootstrapping lower limit confidence interval; BootULCI, bootstrapping upper limit confidence interval; Effect, standardized regression coefficient; SE, standard error.

Additionally, all three indirect and direct paths were significant. Specifically, the first direct pathway showed that professional identity significantly mediated the effect of workplace violence on patient safety, with an effect value of −0.3070. The second direct pathway showed that the effect of workplace violence on patient safety was significantly mediated by professional burnout, with an effect value of −0.1432. The third indirect pathway showed that the effect of workplace violence on patient safety was significantly mediated by both professional identity and burnout, with an effect value of −0.1151. Furthermore, professional identity had a significant direct effect on professional burnout, with an effect value of −0.6160. These results suggest that professional identity and burnout mediate the relationship between workplace violence and patient safety.

## 4 DISCUSSION

This study's primary objective was to determine whether professional identity and burnout mediate the relationship between workplace violence and patient safety among nursing interns. First, the results showed that workplace violence significantly affected patient safety for nursing interns. We also set out to determine whether workplace violence had an indirect negative effect on patient safety through the mediating effect of professional identity and burnout. Finally, professional identity and burnout also had a serial mediation effect on the relationship between workplace violence and patient safety.

The results suggest that an increase in nursing interns' perception of the frequency of workplace violence could directly increase patient safety. This result is consistent with prior studies that link workplace violence to negative patient outcomes (Banda et al., 2016; Graham, 2017). Hassankhani et al. (2018) indicated that exposure to work‐related violence and aggression may increase the risk of negative responses by medical staff, ultimately leading to decreased quality of care. Workplace violence had negative effects on the social integrity, mental health, and professional performance of medical staff. A longitudinal study demonstrated that workplace violence experienced by medical staff is linked to lower quality of care ratings by patients (Arnetz & Arnetz, 2001). This study highlights the significance of preventing workplace violence among nursing interns to build a healthy and safe work environment. Efforts should be made to encourage nursing interns to take part in developing and implementing programs that lower their risk of injury. In addition, our findings support the implementation of “safe hospital” policies and demonstrate the need for zero tolerance of workplace violence among medical staff, including nursing interns, to improve patient safety.

This study demonstrated that professional identity and burnout can mediate the indirect effect of workplace violence on patient safety in nursing interns. On the one hand, the results indicated that the workplace violence experienced by nursing interns created doubts regarding their original choice of becoming nursing students and contributed to a low level of professional identity, which, in turn, decreased patient safety. This finding is consistent with those of prior studies linking workplace violence to lower professional identity, lower job satisfaction, and negative patient outcomes (Blake, 2016; Graham, 2017; Qiu et al., 2019). A phenomenographic study indicated that workplace violence can reduce emergency nurses' professional identity at work and increase burnout and turnover intention, eventually leading to unsafe care (Han et al., 2017).

On the other hand, the results indicated that workplace violence can increase nursing interns' professional burnout, thus affecting patient safety. This mechanism can be explained by the job demands‐resources model, which posits that job demands, such as an unfavourable work environment, high work pressure, and interpersonal conflicts, may result in professional burnout and negatively influence employees' professional performance (Nakagawa et al., 2014). These results are congruent with previous research that shows a negative indirect effect of workplace violence on negative patient outcomes, such as falls and medication errors, because of nurses' lower job satisfaction and intent to leave (Quine, 2001; Roche et al., 2010). Our study's results indicate that concerns about nursing interns' attitudinal or emotional changes, along with workplace violence, cannot be underscored. Exploring how to improve nursing interns' professional identity and reduce professional burnout after experiencing workplace violence may be helpful in increasing the quality of healthcare services.

Finally, in addition to examining the independent mediating role of professional identity and burnout, we also tested whether a chain‐mediating role between workplace violence and patient safety exists. The results of the current study indicated that workplace violence first had a negative correlation with professional identity and then increased professional burnout, which was, in turn, related to reduced nursing interns‐reported patient safety. Regarding the relationship between workplace violence and professional identity, Guan (2017) pointed out that workplace violence can lead to serious consequences, such as reduced work efficiency, frequent absenteeism, and increased occupational turnover (Guan, 2017). This may be because medical staff experiencing workplace violence physically or mentally will hardly concentrate at work and will have difficulties in their relationships with patients. This will lead to nursing interns doubting their original choice as nurses and a low level of professional identity. Regarding the relationship between professional identity and burnout, Zhang, Feng, et al. (2021); Zhang, Zuo, et al. (2021) showed that professional identity is an important influencing factor in turnover intention and burnout. Job satisfaction is associated with an individual's perception and evaluation of the profession, and this perception is influenced by person‐specific values, demands, and expectations. Therefore, nursing interns with a low level of professional identity were less likely to achieve accomplishment from work and have a low level of enthusiasm for work, which in turn increased professional burnout. Finally, this study showed that professional burnout is negatively related to patient safety. A systematic review found that individual professional burnout could help explain variations in safety culture within organizations (Mossburg & Dennison Himmelfarb, 2021).

### 4.1 Implications for research and practice

Based on our results, we suggest that, steps must be taken to prevent workplace violence among nursing interns. The healthcare system should adopt a “safe hospital” policy, in which hospital administrators can provide support when nursing interns experience workplace violence. These findings also indicate that increasing professional identity and decreasing professional burnout may be novel targets for interventions aimed at ensuring patient safety. For example, we can reframe medical education (Cruess et al., 2014), apply stress‐reduction techniques, and provide implications for burnout management to increase professional identity and decrease professional burnout (Santos & Evans, 2020).

### 4.2 Limitations

The current study has some limitations that should be considered. First, our research design was cross‐sectional, which did not allow us to determine the causal relationships between variables. Second, the data we measured on patient safety were nursing intern‐reported, thus it were not objective patient‐outcome data. Finally, other factors such as subjective job stress and professional tasks, which may influence adverse patient outcomes, were not addressed in this study.

## 5 CONCLUSION

Our findings suggest that workplace violence can affect patient safety through decreasing professional identity and increasing professional burnout among nursing interns. Interventions aimed at decreasing workplace violence among nursing interns would be beneficial for professional attitude and patient safety. First, nursing administrative personnel should take action to prevent workplace violence among nursing interns, such as improving nursing interns' communication skills and involvement in workplace violence prevention programs. Moreover, the results suggest that enhancing professional identity and decreasing professional burnout could be a viable target for improving patient safety.

## FUNDING INFORMATION

No external funding.

## CONFLICT OF INTEREST

There are no conflicts of interest involved in the current study.

## PRACTICAL IMPLICATIONS

First, nursing administrative personnel should take action to prevent workplace violence among nursing interns, such as improving nursing interns' communication skills and involvement in workplace violence prevention programs. Moreover, the results suggest that enhancing professional identity and decreasing professional burnout could be a viable target for improving patient safety.

## ETHICAL APPROVAL

The current study was approved by the Ethics Committee of Liaocheng People's hospital (2021098), before the data collection. The attributes, benefits, uses, and disadvantages of the study were explained to all participants, and informed consent was obtained.

## Data Availability

All data generated during this study are included in this published article.
